# Photocurable GelMA Adhesives for Corneal Perforations

**DOI:** 10.3390/bioengineering9020053

**Published:** 2022-01-28

**Authors:** Inês A. Barroso, Kenny Man, Thomas E. Robinson, Sophie C. Cox, Anita K. Ghag

**Affiliations:** School of Chemical Engineering, University of Birmingham, Birmingham B15 2TT, UK; IXP799@student.bham.ac.uk (I.A.B.); k.l.man@bham.ac.uk (K.M.); TER281@student.bham.ac.uk (T.E.R.); S.C.Cox@bham.ac.uk (S.C.C.)

**Keywords:** adhesives, cornea, gelatin

## Abstract

The current treatments for the management of corneal and scleral perforations include sutures and adhesives. While sutures are invasive, induce astigmatism and carry a risk of infection, cyanoacrylate glues are toxic, proinflammatory and form an opaque and rough surface that precludes vision. Consequently, the clinical need for a fast curing and strong tissue adhesive with minimised cytotoxicity and host inflammation remains unmet. In this paper, we engineer a gelatine methacryloyl (GelMA) adhesive that can be crosslinked in situ within 2 min using UV or visible light and a riboflavin (RF)/sodium persulfate (SPS) system. Optical coherence tomography (OCT) images demonstrated that the flowable GelMA adhesive could completely fill corneal wounds and restore the ocular curvature by forming a smooth contour on the ocular surface. Further, ex vivo studies in porcine eyes showed that GelMA bioadhesives exhibited burst pressures that were comparable to cyanoacrylates (49 ± 9 kPa), with the hydrogels exhibiting a transmittance (90%), water content (85%) and storage modulus (5 kPa) similar to the human cornea. Finally, using human dermal fibroblasts, we showed that our GelMA adhesive was non-toxic and could effectively support cell adhesion and proliferation. Taken together, the adhesive’s performance, injectability and ease of administration, together with gelatin’s availability and cost-effectiveness, make it a potential stromal filler or sealant for corneal and conjunctival applications.

## 1. Introduction

Globally, corneal blindness is estimated to affect approximately 23 million people, with an estimated 1.5 million new cases per year [1,2]. Corneal wounds and perforations can arise from infections, traumatic injuries, surgical procedures (e.g., cataract surgery), autoimmune diseases (e.g., ulcerative keratitis) or degenerative disorders [3,4]. Full-thickness corneal injuries can leak and allow the ingress of pathogens into the eye. Therefore, given the risk of ocular morbidity and vision loss, corneal perforations are considered surgical emergencies [5]. Currently, the sealing of corneal wounds remains challenging for surgeons and sutures are still the gold standard treatment for the closure of ocular wounds due to their high tensile strength [5,6]. However, sutures are time consuming to position, inflict additional trauma to the tissues, can act as a nidus for infection, induce astigmatism and often require secondary removal procedures [2,7,8]. 

In contrast, tissue adhesives are an appealing alterative to the use of sutures. Tissue adhesives exhibit lower infection rates, can be delivered through smaller applicators and form a continuous seal, adapting to the wound morphology and distributing the load, thereby decreasing scarring [9,10]. The development of an effective and safe tissue adhesive could reduce surgical and hospitalisation time, lower the incidence of complications and reduce leakage from wounds [2]. Specifically, the ideal material to repair corneal perforations must be optically clear to allow vision, exhibit strong adhesive properties to the naturally moist ocular surface, provide healing enhancement without scarring or vascularisation and have a rapid in situ crosslinking after application [2,10,11,12,13,14]. Nowadays, ocular adhesives can be divided into two main groups: naturally derived (e.g., fibrin-based) and synthetic adhesives (e.g., cyanoacrylate and PEG-based) [4].

In ocular surgery, fibrin sealants have been used to seal corneal perforations (with and without an amniotic membrane), lens capsule lacerations and to repair leaking blebs [4,15]. Fibrin glues are biodegradable, easy to apply and show minimal toxicity together with improved comfort. The main drawbacks of these bioinspired glues are the potential risk of disease transmission and fast degradation in vivo (within days to weeks), which limits its use and/or requires re-application. Currently, fibrin-based adhesives are mainly used for amniotic membrane fixation [4,15,16]. Although cyanoacrylate-based adhesives (CAs) are not FDA approved for ophthalmic use, these adhesives have been used off-label in the eye for several decades [17,18]. In ocular surgery, CAs are reported to be toxic, proinflammatory and are associated with many postoperative complications, such as keratitis [19,20], unintentional entry into the anterior chamber [21,22], corneal neovascularization and scar formation [4,16,23]. Nonetheless, CAs remain a useful tool in the treatment of ocular surgical emergencies due to their quick preparation, easy application and effective and secure performance. Another class of synthetic tissue adhesives are based on the FDA-approved polymer poly(ethylene glycol) (PEG). PEG-based adhesives are safe, well tolerated, elastic and biodegradable, work independently of the clotting cascade, do not carry risks of disease transmission and some formulations remain in situ longer than fibrin sealants [24,25]. ReSure (Ocular Therapeutix, Bedford, MA, USA) and OcuSeal (BD Medical, Le Pont de Claix, France) are approved for sealing corneal incisions. However, despite being useful to reinforce suture sealing and close corneal incisions, these adhesives are not appropriate to function as stromal fillers due to poor mechanical properties, high swelling rates and short in vivo retention [24,26,27]. 

Over the last decade, several polymers have been studied in an effort to develop novel tissue adhesives, such as alginate [28,29], chondroitin sulphate [30], chitosan [28,31,32], tropoelastin [33], gelatine [11,13,34,35,36,37], poly(acrylamide-methyl acrylate-acrylic acid) [38] and N-iso-propylacrylamide copolymerized with butylacrylate [39]. However, currently the clinical needs are still unmet with no materials specifically approved for the filling and repair of corneal defects [40].

Gelatine is a promising biomaterial for the repair of corneal defects given its availability, cost-effectiveness, water solubility and presence of both cell-binding sites (e.g., RGD peptide sequence) and matrix metalloproteinase (MMPs) motifs that promote cellular adhesion and allow enzymatic degradation, respectively [41,42]. However, it requires modification to enable mechanical tailoring via chemical crosslinking. Once methacrylic/methacryloyl groups (GelMA) are introduced, these bioadhesives can be photocrosslinked under physiological conditions within seconds. The final adhesive properties can be tailored by changing the initial gel strength of raw gelatine (Bloom factor), the degree of methacryloyl functionalisation (DF%), polymer concentration, light characteristics and time. Recently, GelMA-based adhesives photocrosslinked using type I photoinitiators, such as LAP (Lithium phenyl-2,4,6 trimethylbenzoylphosphinate) [36] and Irgacure-2959 [11,13,35,43], or type II photoinitiators, such as ruthenium/SPS [34], RF/TEA (triethanolamine) [44] and Eosin Y/VC(N-vinylcaprolactam)/TEA [37,45,46,47,48], have been investigated for different applications. In ocular surgery, there have been studies on the development of GelMA adhesives/hydrogels to be photocrosslinked in situ [13,37,45,48] or implanted after curing [35]. 

In this paper, we develop an optically clear and injectable gelatine-based adhesive that can seal corneal defects. In this work, we use RF as a photoinitiator since it is already being used in corneal crosslinking (CXL), an FDA-approved procedure to treat patients with post laser in situ keratomileusis (LASIK) ectasia and progressive corneal thinning (keratoconus) [49,50]. Furthermore, RF is a naturally occurring photosensitive molecule (vitamin B2) and absorbs light in both the UV (365 nm) and visible range [51]. In this study, GelMA bioadhesives of different polymer concentrations are crosslinked using UV or visible light and a photoinitiator system composed by RF and SPS: 15 (*w*/*v*)% and 20 (*w*/*v*)% GelMA crosslinked with visible light (referred as 15% and 20%, respectively) and 20 (*w*/*v*)% GelMA crosslinked with UV light (20% UV). 

**Figure 1:**
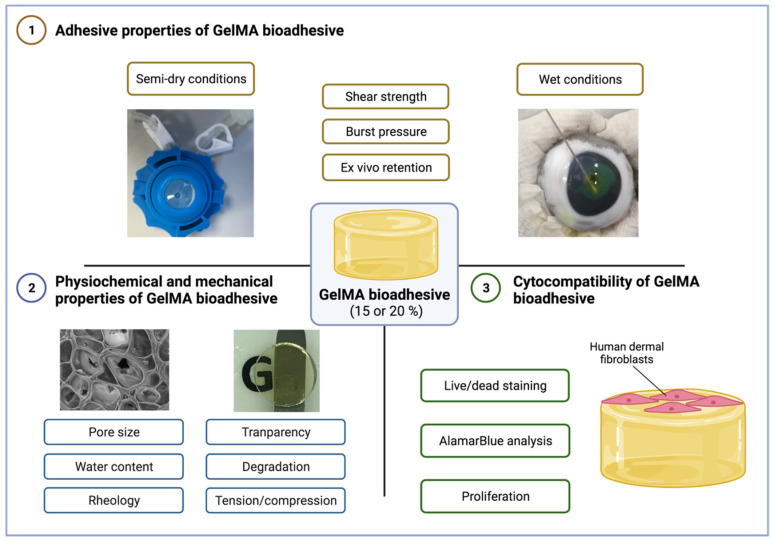
Schematic outline investigating the properties of GelMA bioadhesives. Figure created with BioRender.com (last accessed 8 November 2021).

To our knowledge, this is the first work investigating GelMA hydrogel formation via riboflavin/SPS mediated photocrosslinking. As shown in Figure 1, the physicochemical, adhesive, and biological properties of GelMA bioadhesives are characterised. Briefly, we conclude that GelMA bioadhesives can be injected in situ and rapidly seal full-thickness corneal perforations (≤2 min). The adhesives restored the corneal curvature by forming a smooth and transparent seal, while withstanding intraocular pressures comparable to Histoacryl (33–50 kPa). The versatility of the semi-synthetic bioadhesives developed in this study to function on dry, semi-dry and wet substrates make them suitable to be applied not only for sealing corneal and conjunctival lacerations, but also in different physiological conditions (e.g., skin).

## 2. Materials and Methods

### 2.1. GelMA Synthesis

Briefly, a 10% (*w*/*v*) gelatine type A solution (porcine skin, 300 bloom strength, Sigma-Aldrich, Gillingham, UK) was dissolved in phosphate-buffered saline (PBS, Sigma-Aldrich, Gillingham, UK) at 60 °C for 30 min. Methacrylic anhydride (MA, Sigma-Aldrich, Gillingham, UK) was added to the gelatine solution to a final concentration of 6% (*v*/*v*) and left to react for 2 h at 50 °C under vigorous stirring. After the reaction period, the solution was transferred to 50 mL tubes and the unreacted MA was partially removed by centrifugation at 3500 rpm for 5 min at room temperature. The solution was then dialysed against distilled water for 7 days at 37 °C using 12–14 kDa cut-off dialysis tubes (Thermo Scientific, Paisley, UK). After dialysis, the GelMA solution was diluted to 2% (*w*/*v*) and the pH adjusted to 7.4 using 1 mM sodium hydroxide solution (Sigma-Aldrich, Gillingham, UK). Lastly, GelMA solutions were lyophilised for 2 days to generate a white porous foam, which was stored at −80 °C until further use.

### 2.2. Degree of Functionalisation

The degree of functionalisation (DF%) was quantified by proton nuclear magnetic resonance (^1^H-NMR). Briefly, 5 mg of GelMA was dissolved in 600 μL deuterium oxide (Sigma-Aldrich, Gillingham, UK). The ^1^H-NMR spectra of raw gelatine and GelMA were recorded using a 400 MHz spectrometer (Brucker, Billerica, MA, USA). Baseline and phase correction were applied before integrating the peaks of interest with Topspin software (Brucker, Billerica, MA, USA). The phenylalanine signal (7.2–7.5 ppm) was used as the internal reference to normalise the amine signals (3.13–3.22 ppm) of lysine [52]. As MA reacts with gelatine through the primary amines on lysine residues, the extent of substitution was calculated by normalisation to the number of free amino groups of raw gelatine. The degree of functionalisation was calculated using the following equation:

(1) $$\mathrm{DF}(\%)=\frac{\mathrm{lysine}\;\mathrm{integration}\;\mathrm{signal}\;\mathrm{of}\;\mathrm{GelMA}}{\mathrm{lysine}\;\mathrm{integration}\;\mathrm{signal}\;\mathrm{of}\;\mathrm{Gelatin}}\times 100$$

### 2.3. Preparation of GelMA Bioadhesives 

GelMA prepolymer solutions were prepared by dissolving an appropriate amount of polymer in PBS at 60 °C for 30 min. Fresh stock solutions of 40 mM phosphated riboflavin (RF, Sigma-Aldrich, Gillingham, UK) and 456 mM sodium persulfate (SPS, Sigma-Aldrich, Gillingham, UK) in PBS were prepared. The photoinitiator system RF/SPS was added to the GelMA solution to a final concentration of 2/20 mM and the resulting solution was vigorously mixed at 60 °C. The hydrogel precursor solutions were photocured using 30 mW/cm^2^ UV light with an OmniCure S1500 (Excelitas Technologies, Ontario, Canada) with a 365 nm filter or using 100 mW/cm^2^ visible light (Knightsbridge FLF Floodlight, RS, Corby, UK). Briefly, 200 μL of GelMA prepolymer solution was pipetted onto a 12 mm cylindrical mould and then exposed to light for 2 min. In this work, three different formulations were tested: 15 (*w*/*v*)% and 20 (*w*/*v*)% GelMA crosslinked with visible light (referred as 15% and 20%, respectively) and 20 (*w*/*v*)% GelMA crosslinked with UV light (20% UV).

### 2.4. Adhesive Properties

#### 2.4.1. Ex Vivo Burst Pressure

**Figure 2:**
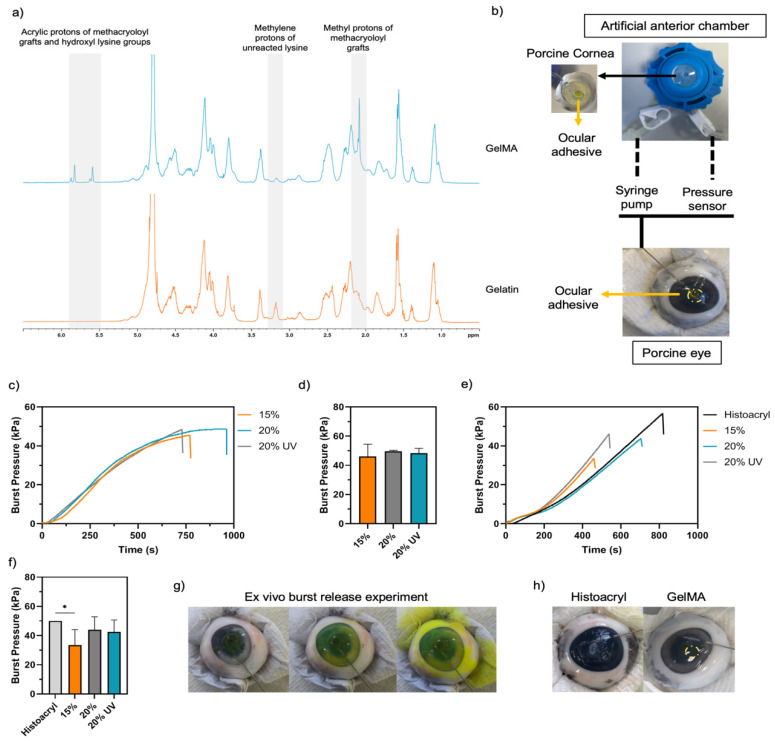
(**a**) ^1^H-NMR spectra of GelMA (top) and unfunctionalized gelatine (bottom). Ex vivo burst pressure of GelMA adhesives. (**b**) Schematic of the ex vivo burst pressure measurement set-up. A Barron artificial anterior chamber was used to assess the GelMA adhesion in semi-dry conditions. A porcine cornea with a 2 mm full-thickness injury was mounted on the chamber. To simulate the in vivo conditions, a full-thickness injury in a porcine eyeball was sealed with GelMA adhesive. In both systems, a fluorescein solution was injected using a syringe pump until bursting. (**c**) Representative pressure curves of the studied groups when the cornea was mounted in the artificial anterior chamber. (**d**) Mean burst pressure of GelMA adhesives in the artificial anterior chamber (*n* = 8). (**e**) Representative pressure curves of the studied groups and Histoacryl glue in the porcine eyeball. (**f**) Mean burst pressure of GelMA adhesives and Histoacryl glue in the porcine eyeball (*n* = 8). (**g**) Porcine eyeball before, during and after the burst pressure experiment. (**h**) Appearance of full-thickness corneal injuries sealed with Histoacryl and GelMA. Data presented as mean value ± SD (* *p* < 0.05).

The ability of engineered adhesives to seal corneal perforations was assessed on wet and dry conditions. Porcine eyes were chosen as an ex vivo model due to their availability, which allowed freshly enucleated eyes to be obtained from a local butcher on a daily basis. To study adhesion on wet conditions, a full-thickness incision was created on the porcine eyeball using a 2 mm biopsy punch and 20 μL of GelMA adhesive or Histoacryl (control group, B. Braun Surgical S.A., Barcelona, Spain) was added to the perforation and cured in situ. Then, the sealed eyes were connected to a pressure testing system (Figure 2b) through the insertion of a 27-gauge needle into the anterior chamber of a porcine eyeball. Finally, a syringe pump (SINO SN-50F66, SINO MDT, Shenzhen, China) injected PBS mixed with fluorescein (Sigma-Aldrich, Gillingham, UK) into the anterior chamber at a rate of 20 mL/h, while a wireless pressure sensor (PASCO, Roseville, CA, USA) recorded the intraocular pressure. The test was stopped at adhesive failure or when the pressure reached 50 kPa (*n* = 8). The burst pressure on dry conditions was studied by mounting a porcine cornea with a 2 mm full-thickness perforation on a Barron artificial anterior chamber (AAC, Katena, Parsippany, New Jersey, USA) (Figure 2b). The burst pressure was measured as previously described (*n* = 8).

#### 2.4.2. Lap Shear Strength

**Figure 3:**
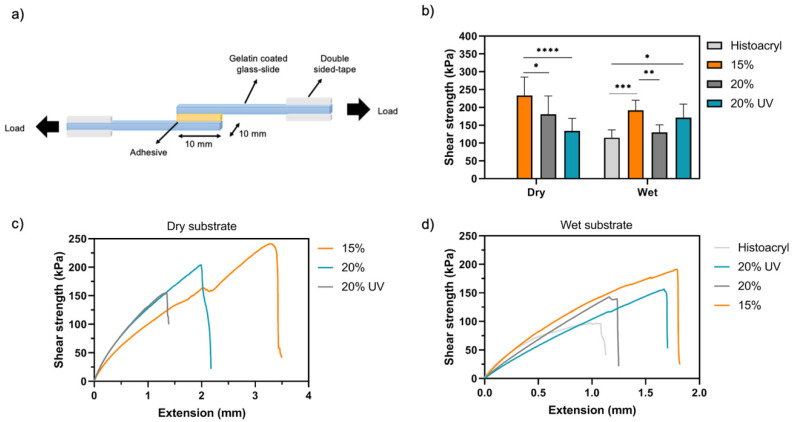
In vitro shear strength of GelMA adhesives: (**a**) Schematic of the lap shear strength test and (**b**) mean shear strength of GelMA bioadhesives and Histoacryl in wet and dry conditions. (**c**,**d**) Representative stress–strain curves of GelMA hydrogels and Histoacryl in dry and wet substrates, respectively. Data presented as mean value ± SD (* *p* < 0.05, ** *p* < 0.01, *** *p* < 0.001, **** *p* < 0.0001).

The shear strength of GelMA adhesives and Histoacryl (control group, B. Braun Surgical S.A., Barcelona, Spain) was tested according to the modified ASTM F2255-05 standard for tissue adhesives using an Instron 5542 mechanical tester (Instron, Norwood, MA, USA) with a 2 kN load cell. As substrate, two pieces of glass slides (50 × 10 mm) were coated with 20% (*w*/*v*) gelatine solution (porcine, 300 bloom, Sigma-Aldrich, Gillingham, UK) at 60 °C and left to dry at room temperature overnight. Then, 20 μL of prepolymer solution was crosslinked between two gelatine-coated glass slides (total area = 100 mm^2^). The two glass slides were placed between two pieces of double-sided tape, fastened to the instrument’s tension grips and extended with a strain rate of 2 mm/min (Figure 3a). The shear strength of the adhesives was calculated at the detachment point on dry and wet substrates (*n* = 7).

### 2.5. Transparency

Light absorbance of hydrogels in the visible range was measured using a microplate reader (Spark Multimode, Tecan, Männedorf, Switzerland). The measurements were carried out in triplicate using GelMA hydrogels in PBS (diameter = 12 mm, thickness = 1.2 mm), with pure PBS serving as a blank control. Subsequently, the transmittance was calculated using the following equation:


(2) $$\mathrm{Transmittance}\;(\%)=10^{2-\mathrm{Absorbance}}$$

### 2.6. Swelling Properties

Immediately after preparation, three samples (diameter = 12 mm, thickness = 1.2 mm) from each condition were freeze dried ($m_{t0,\mathrm{dry}}$) and three samples were hydrated in PBS and placed in an incubator at 32 °C. After 24 h, the samples were blotted dry using a filter paper and the swollen weight ($m_{\mathrm{swollen}}$) was measured. Finally, the swollen hydrogels were lyophilised and weighed to determine the dry sample weight ($m_{\mathrm{swollen},\mathrm{dry}}$). The mass loss is a measurement of the macromers not crosslinked in the hydrogel network [53]. The water content and mass loss were calculated according to the following equations:

(3) $$\mathrm{Water}\;\mathrm{content}\;\left(\%\right)=\frac{m_{\mathrm{swollen}\;}-m_{t0,\mathrm{dry}}}{m_{\mathrm{swollen}}}\times 100$$

(4) $$\mathrm{Mass}\;\mathrm{loss}=\frac{m_{t0,\mathrm{dry}\;}-m_{\mathrm{swollen},\mathrm{dry}}}{m_{t0,\mathrm{dry}}}\times 100$$

### 2.7. Enzymatic Degradation

Disc-shaped hydrogels (diameter = 6 mm, thickness = 2 mm) were prepared as previously described. After photocrosslinking, five samples of each group were immediately freeze dried to calculate the initial weight. To determine the in vitro degradation, 5 samples of each group were placed in 1.5 mL tubes with 1 mL of 1 U/mL collagenase II in PBS (Thermo Fisher Scientific, Waltham, MA, USA) or in 1 mL PBS (control group) and incubated at 37 °C for 1, 3 and 7 days. At the end of the incubation period the hydrogels were lyophilised. The degradation was calculated using the following equation:

(5) $$\mathrm{Degradation}\;\left(\%\right)=\frac{m_{t0,\mathrm{dry}\;}-m_{\mathrm{dry}}}{m_{\mathrm{dry}}}\times 100$$

### 2.8. Micro Computed Tomography

Freeze-dried hydrogels were scanned using a Skyscan 1172 (Bruker, Billerica, MA, USA) with 80 kV beam voltage and 100 μA current, 140 ms exposure per projection, 12.99 μm pixel size, rotation step 0.6° and 4 frame averaging. The same parameters were used for all scans. The XY projections were reconstructed into a 3D model using NRecon (Bruker). A cylindrical volume of interest was taken in the centre of each sample, excluding any outside space. A binary threshold was then applied to this volume of interest to separate polymer material and air in the pores of the sample. The pore size distribution was then calculated using the 3D analysis software in CTAn (Bruker). Sample reconstructions were visualized in 3D using CTVox (Bruker). The experiments were conducted in triplicate.

### 2.9. Scanning Electron Microscopy

Scanning electron microscopy (SEM) was used to assess the morphology of the surface of the freeze-dried hydrogels. All samples (diameter = 6 mm, thickness = 2 mm) were mounted onto aluminium stubs using carbon tape and gold sputter coated. Images were captured using a TM3030Plus benchtop SEM (Hitachi High Technologies, Schaumburg, IL, USA) at an electron acceleration voltage of 15 kV.

### 2.10. Rheological Characterisation

The viscoelastic properties of the hydrogels were investigated via rheometry. The storage modulus and loss modulus of freshly prepared hydrogels were measured in oscillatory mode at 32 °C using a plate–plate geometry and a 1.2 mm gap between plates (Kinexus Pro+, Malvern Instruments, Malvern, UK). A frequency sweep was conducted between 0.01–10 Hz at 0.5% shear strain. The experiments were conducted in triplicate. The damping factor (tan δ) was calculated using the following equation:

(6) $$\tan\;\delta =\frac{G^{\prime \prime }}{G^{\prime }}$$

### 2.11. Mechanical Characterisation

#### 2.11.1. Compressive Modulus

Cyclic testing was performed using an Instron 5542 mechanical tester (Instron, Norwood, MA, USA) with a 2 kN load cell. Cylindrical hydrogels were prepared as previously described and incubated in PBS for 4 h prior to testing. The dimensions of the samples were determined using a digital calliper. Compressive tests were performed at a rate of 1 mm/min up to a maximum strain of 60% of the original height by performing 8 cycles of loading and unloading. The compressive strain (mm) and load (N) was recorded using Bluehill 3 software (Instron, Norwood, MA, USA). The compressive moduli were calculated from the slope of the linear region on the stress (kPa) versus strain (mm/mm) curves. Samples were tested in triplicate for each condition.

#### 2.11.2. Tensile Modulus

Uniaxial tensile testing was performed using an Instron 5542 mechanical tester (Instron, Norwood, MA, USA) with a 100 N load cell. Hydrogels were prepared as previously described in dog-bone shaped moulds (width = 4 mm, gauge length = 10 mm) and incubated in PBS for 4 h prior to testing. After measuring the swollen sample dimensions using a digital calliper, hydrogels were placed between two pieces of double-sided tape, fastened to the instrument’s tension grips and extended at a rate of 2 mm/min until failure. The load (N) and tensile strain (mm) were measured using Bluehill 3 software and the tensile modulus was calculated from the slope of the stress–strain curves. The tensile strength and maximum elongation were obtained at point of failure. At least five samples were tested for each condition. 

### 2.12. Ex Vivo Retention Time 

Freshly enucleated porcine eyes were obtained from a local butcher. After the creation of a 5 mm corneal defect with a biopsy punch (50% deep), 10 μL of prepolymer solution were applied to the defect site and photocrosslinked. OCT imaging was used to assess the ability of the engineered adhesives to fill the corneal defect and adhere to the corneal stroma immediately after photocrosslinking (t_0_) and to study the dimensional stability and retention time of the hydrogels after incubation. Samples were imaged with a Telesto II SD-OCT System (Thorlabs, Lübeck, Germany) at t_0_ and after 1, 3 and 7 days of incubation in 20 mL of PBS with 5% penicillin/streptomycin (Sigma-Aldrich, Gillingham, UK) at 4 °C. 

### 2.13. In Vitro Cytocompatibility of GelMA Hydrogels

Human dermal fibroblasts (HDFs, ATCC, USA Passage number 9) were cultured in Dulbecco’s modified eagle medium (DMEM, Sigma-Aldrich, Gillingham, UK) and supplemented with 10% foetal bovine serum (Sigma-Aldrich, Gillingham, UK), 1% penicillin/streptomycin (Sigma-Aldrich, Gillingham, UK) and 2 mM L-glutamine (Sigma-Aldrich, Gillingham, UK). All cultures were maintained in tissue culture treated polystyrene flasks at 37 °C with 5% CO_2_ in a humidified incubator and passaged at 80% confluency.

#### 2.13.1. 2D Cell Seeding on GelMA Gels

GelMA pre-polymer solutions were prepared, UV sterilised and photocrosslinked as previously described in 48-well suspension cell culture plates (Sarstedt, Nümbrecht, Germany). After curing, 100 μL of DMEM was added to the hydrogels to prevent dehydration. One hour before cell seeding, the hydrogel matrices were partially dried to potentiate cell penetration. Then, 60 μL of cell suspension was added dropwise onto each hydrogel (9 × 10^3^ cells per scaffold) and samples were incubated for 1 h at 37 °C with 5% CO_2_ to bolster cell adhesion. After 1 h, DMEM was carefully added to the wells without disturbing the cell-laden hydrogels [54]. The culture medium was changed every two days. 

#### 2.13.2. Cell Viability

Cell viability was evaluated using a live/dead assay (Thermo Fisher Scientific, Waltham, MA, USA) following the manufacturer’s protocol. Briefly, SYTO 10 green fluorescent nucleic acid stain (2 μL/mL) and ethidium homodimer-2 nucleic acid stain (2 μL/mL) were diluted in PBS to form the staining solution. At each time point, the samples were incubated with the staining solution for 15 min in the dark at 37 °C. The staining solution was removed, and the samples were washed twice with PBS. The cell-seeded hydrogels were imaged with a confocal scanning microscope (Zeiss LSM710, Carl Zeiss, Jena, Germany). 

#### 2.13.3. Metabolic Activity

Alamar Blue assay (Thermo Fisher Scientific, Waltham, MA, USA) was used to determinate the relative metabolic activity according to the manufacturer’s directions. In short, the samples were incubated with 0.5 mL of 10% Alamar Blue reagent in culture medium for 4 h at 37 °C with 5% CO_2_. Thereafter, 50 μL of cell culture medium were transferred to a 96-well plate (Corning, Corning, NY, USA) and the fluorescence intensity was measured with a microplate reader (Spark Multimode, Tecan, Männedorf, Switzerland) using an excitation wavelength of 540 nm and an emission wavelength of 590 nm. Acellular hydrogels were used as a negative control and their fluorescence was subtracted from the same group seeded hydrogels to account for the background (*n* ≥ 4). 

#### 2.13.4. Cell Proliferation

DNA content was quantified using the Quant-iT PicoGreen DNA assay (Life Technologies, Paisley, UK). Briefly, cell-laden hydrogels were lysed in 0.1% TritonTM X-100 in PBS (Sigma-Aldrich, Gillingham, UK) via performing freeze–thaw cycles between 37 °C and –80 °C. After spinning down the samples at 300 g for 5 min to remove non-genomic material, 10 μL of cell lysate was added to 90 μL of TE (10 mM Tris-HCl, 1 mM EDTA) buffer in a 96-well plate (Corning, Corning, NY, USA). A total of 100 μL of PicoGreen was added to all samples and then incubated for 5 min. The fluorescence intensity was then measured with a microplate reader (Spark Multimode, Tecan, Männedorf, Switzerland) at an excitation and emission wavelength of 480 and 520 nm, respectively. 

### 2.14. Statistical Analysis

For each experiment, at least three samples were tested. One or two-way analysis of variance (ANOVA) and Tukey’s test were used to determine statistically significant differences with GraphPad Prism 8.0 software (GraphPad Software, San Diego, CA, USA). The level of significance was set at *p* < 0.05. Data are presented as mean value ± standard deviation (SD).

## 3. Results

### 3.1. Synthesis of GelMA Bioadhesives

^1^H-NMR spectroscopy was used to confirm the methacryloyl functionalisation of GelMA (Figure 2a). The ^1^H-NMR of GelMA displays peaks in the 5–6 ppm range and at ~1.8 ppm, corresponding to the acrylic protons of methacryloyl of lysine and hydroxyl lysine groups and the methyl proton of methacryloyl grafts, respectively [55,56,57]. These groups are absent in the spectra of unmodified gelatine, indicating the covalent functionalisation of gelatine with the methacryloyl groups. The marked decrease in the free lysine signal at 3.1–3.2 ppm indicated that the DF% was 60% after 2 h reaction with 6% (*v*/*v*) MA.

### 3.2. Adhesive Properties of GelMA Bioadhesives

The success of a bioadhesive depends on its adhesion and retention at the wound site [29]. In this study, burst pressure and lap shear tests were performed to characterise the GelMA bioadhesive properties in semi-dry and wet conditions. A cyanoacrylate-based adhesive (Histoacryl, B. Braun Surgical S.A., Barcelona, Spain) was used as a positive control due to its off-label application in surgical emergencies involving corneal perforations. To quantitively assess the mechanical adhesion of GelMA bioadhesives to the corneal tissue under liquid pressure, GelMA formulations were tested using two different set-ups: an artificial anterior chamber (AAC) and a porcine eye (Figure 2b,g). In the first method, after making a 2 mm full-thickness central injury, the cornea is removed with a scalpel and mounted on the AAC. As only the cornea is mounted on the AAC, this method tests the corneal adhesive performance in a semi-dry environment. However, since liquids at the wound–adhesive interface are a barrier to strong surface bonding and, in case of a corneal perforation, the eye leaks aqueous humour, the performance of GelMA bioadhesives was also studied in the porcine eyeball with a full-thickness perforation. This experimental setup is closer to the real situation and represents the worst-case scenario. In both systems, a fluorescein solution was progressively injected until bursting or reaching the pump’s maximum pressure of 50 kPa. The average burst pressure of GelMA bioadhesives on the AAC was between 46 ± 8 and 50 ± 1 kPa. The difference was not statistically significant between the groups (*p* > 0.05) (Figure 2c,d). As expected, the burst pressures obtained when sealing corneal injuries on the porcine eyeball were lower than in the AAC, with 15%, 20% and 20% UV GelMA bioadhesives exhibiting burst pressures of 33 ± 11, 49 ± 9 and 48 ± 8 kPa, respectively, while Histoacryl had a burst pressure superior to 50 kPa (Figure 2e,f). However, only 15% GelMA and the control were statistically significant (*p* < 0.05). Despite showing high adhesive strength on wet substrates, Histoacryl showed no adhesion onto semi-dry corneal tissue. Furthermore, when this glue was used an opaque and rough patch formed, while GelMA formed a smooth and transparent seal that could restore the curvature of the cornea (Figure 2h). Finally, we observed that the bioadhesive failure position was generally on the interface between the hydrogel and the cornea, suggesting that the adhesion force is lower than the adhesive strength.

Next, lap shear tests were used to investigate the adhesion strength of GelMA bioadhesives to wet and dry substrates (Figure 3a). In line with the results obtained in the burst pressure experiment, when a liquid layer is present on the interface between the substrate and the adhesive, lower shear strengths were obtained for all the groups tested (Figure 3b). However, the minimum strength at break was around 130 kPa for both conditions tested. More specifically, the highest shear strength on dry conditions was obtained for 15% (233 ± 52 kPa), which was significantly higher than 20% (181 ± 51 kPa, *p* < 0.05) and 20% UV (134 ± 35 kPa, *p* < 0.0001). Histoacryl failed to glue the dry gelatine-coated glass slides (Figure 3b,c). On a wet substrate, Histoacryl exhibited a shear strength of 115 ± 22 kPa, which was significantly lower than the results obtained for 15% (192 ± 28 kPa, *p* < 0.001) and 20% UV (171 ± 38 kPa, *p* < 0.05) (Figure 3b,d). Finally, the GelMA bioadhesive failure was caused by hydrogel detachment from the gelatine coating as residual GelMA could be found on one side of the gelatine-coated glass slide after the test (delamination).

### 3.3. Physicochemical Characterisation of GelMA Bioadhesives

**Figure 4:**
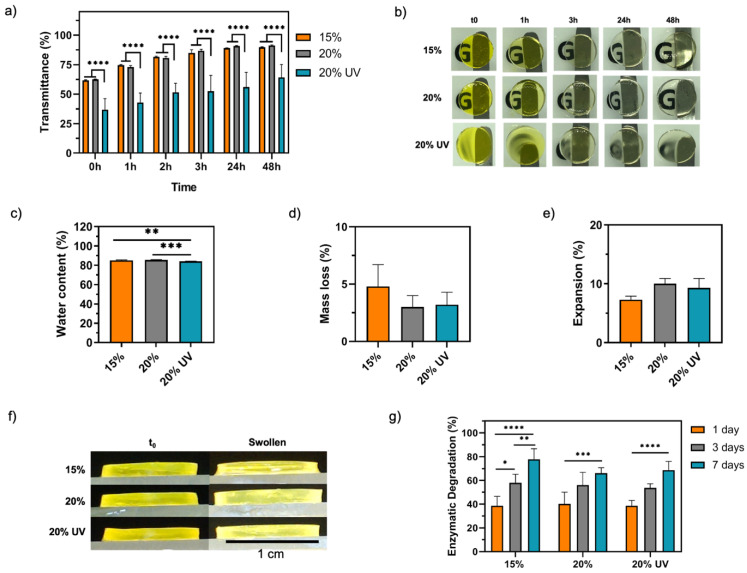
In vitro characterisation of GelMA bioadhesives. GelMA hydrogels transmittance in the visible range: (**a**) average optical transmittance and (**b**) optical transparency of the hydrogels overtime. Hydrogel diameter = 1 cm. Swelling properties of GelMA hydrogels in PBS at 32 °C: (**c**) equilibrium water content (%), (**d**) mass loss (%), (**e**) expansion (%) in PBS and (**f**) lateral image of the hydrogels after photocrosslinking (t_0_) and after 24 h incubation in PBS (swollen). (**g**) In vitro degradation of GelMA hydrogels incubated in 1 U/mL collagenase II at 32 °C for 1, 3 and 7 days. Data are presented as mean value ± SD (* *p* < 0.05, ** *p* < 0.01, *** *p* < 0.001, **** *p* < 0.0001).

The optical transparency of GelMA bioadhesives over time is shown in Figure 4a. After photocrosslinking, 15 and 20% GelMA adhesives were macroscopically clear and lightly yellow, with an optical transmittance of ~62 ± 0.5% (Figure 4b). As riboflavin leaches out from the matrices, the transmittance of GelMA adhesives progressively increased and, after 3 and 24 h, it was approximately 86 ± 0.5% and 91 ± 0.5%, respectively, for both 15 and 20% GelMA adhesives. However, 20% UV samples were not optically clear, showing a maximum transparency of 64.2 ± 11% (Figure 4a,b). The optical transmittance of 20% UV was significantly lower than 15 and 20% at all time points (*p* < 0.0001). 

To determine the water content, mass loss and the percentage of expansion, 15, 20 and 20% UV GelMA bioadhesives were incubated in PBS at the ocular temperature for 24 h. The water content was shown to be higher than 84% throughout all groups studied; however, 20% UV (84.1 ± 0.3%) displayed a significantly lower water content than 15% (85.1 ± 0.5%, *p* < 0.01) and 20% (85.5 ± 0.3%, *p* < 0.001) samples (Figure 4c). Moreover, regardless of polymer concentration or light source used, no significant differences were observed in the percentage of uncrosslinked monomers within the hydrogel network (mass loss = 3–5%) (Figure 4d). This result, together with the fact that the material was easily handled in the swollen state (Figure 4b) and no residual uncured solution could be observed in the mould after curing, suggest complete crosslinking [53]. The expansion of GelMA bioadhesives in PBS was not statistically different between groups, despite ranging from 7.3 ± 0.6% to 10.0 ± 0.9% for 15 and 20% GelMA bioadhesives, respectively (*p* > 0.05) (Figure 4e). Finally, as shown in Figure 4f, GelMA bioadhesives displayed minimal volume changes and high dimensional stability in the swollen state. 

The susceptibility of GelMA bioadhesives to enzymatic degradation was studied in vitro by incubating the hydrogels in 1 U/mL collagenase II for up to 7 days. As shown in Figure 4g, our results show that there was a time-dependent increase in degradation. After 1 and 2 days, the GelMA bioadhesive degradation was around 39% and 55%, respectively. Although no significant differences were observed between the studied groups, after 7 days, 15% bioadhesives (78 ± 9%) showed a degradation that was around ~10% higher than 20% (66 ± 5%) and 20% UV (68 ± 7%) samples.

**Figure 5:**
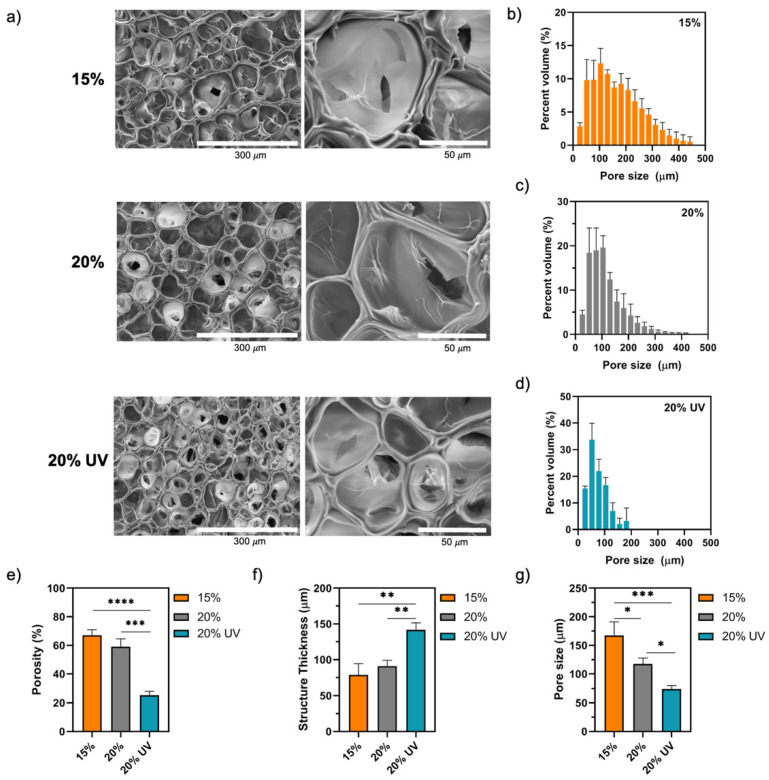
GelMA bioadhesives morphology and microstructure analysis after freeze drying. (**a**) Representative SEM images of GelMA scaffolds at different magnifications. Quantitative analysis of porosity by micro-CT: (**b**–**d**) pore size distribution in the hydrogels, (**e**) total porosity, (**f**) mean pore wall thickness and (**g**) mean pore size. Data are presented as mean value ± SD (* *p* < 0.05, ** *p* < 0.01, *** *p* < 0.001 and **** *p* < 0.0001).

The microstructure of GelMA bioadhesives was investigated using SEM and micro-CT analysis. SEM images of the adhesive’s surface revealed a uniform and interconnected pore structure for all studied groups (Figure 5a). Since SEM images suggest that 20% UV bioadhesives have a smaller pore size than 15 and 20% samples, micro-CT was subsequently used to investigate the internal structure of the bioadhesives. As shown in Figure 5b–d, the pore size distribution was different between the studied groups. The narrower size distribution observed for 20% bioadhesives suggests a negative correlation between polymer concentration and size distribution. Likewise, the light wavelength and intensity used also seem to affect size distribution as 20% UV bioadhesives showed a narrower size distribution compared to 20%. We observed that, for 20% samples, 19 ± 5% and 20 ± 2% of the pores had 50 and 100 µm, respectively, while the prevalence of 50 and 100 µm pores in 20% UV adhesives was 35 ± 5% and 16 ± 3%, respectively (Figure 5c,d). As shown in Figure 5e, the total porosity of 15, 20 and 20% UV bioadhesives was 67 ± 4, 59 ± 6 and 25 ± 3%, respectively. The porosity of 20% UV samples was significantly lower than 15% (*p* < 0.0001) and 20% (*p* < 0.001). Likewise, the structure thickness of 20% UV samples (142 ± 10 µm) was significantly higher than 15% (79 ± 16 µm, *p* < 0.01) and 20% (91 ± 8 µm, *p* < 0.01) bioadhesives (Figure 5f). Finally, the average pore size of 15, 20 and 20% UV samples was significantly different and equal to 167 ± 24, 118 ± 11 and 74 ± 6 µm, respectively (Figure 5g).

**Figure 6:**
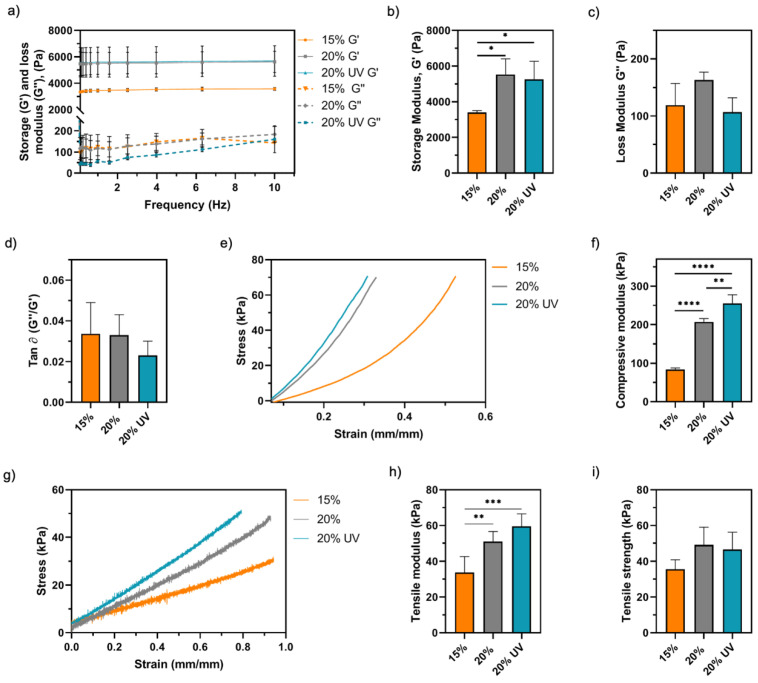
Rheological properties of GelMA bioadhesives: (**a**) frequency sweep, (**b**) storage modulus (G′), (**c**) loss modulus (G″) and (**d**) damping factor (tan δ). Cyclic compressive mechanical testing of GelMA hydrogels: (**e**) representative compressive stress–strain curves and (**f**) compressive moduli. Tensile mechanical testing of GelMA hydrogels: (**g**) representative tensile stress–strain curves, (**h**) tensile moduli and (**i**) maximum tensile strength. Data are presented as mean value ± SD (* *p* < 0.05, ** *p* < 0.01, *** *p* < 0.001, **** *p* < 0.0001).

The viscoelastic properties of GelMA bioadhesives were studied via oscillatory rheometry. Storage (G’) and loss (G’’) modulus represent the elastic and reversible response of the material and the viscous and irreversible rearrangement of its polymeric structure, respectively [58,59]. As shown in Figure 6a,b, a positive correlation between G’ and the polymer concentration was observed. A total of 15% bioadhesives (3.4 ± 0.1 kPa) had a 55% lower G’ than 20% (5.5 ± 0.9 kPa, *p* < 0.05) and 20% UV (5.2 ± 1.1 kPa, *p* < 0.05). The G’’ values were not statistically different between groups, ranging from 107 and 163 kPa (Figure 6c). Moreover, the damping factor (G’’/G’) was lower than 0.03 for all the samples (Figure 6d). Then, the mechanical performance of GelMA bioadhesives was assessed by compressive and tensile mechanical testing (Figure 6e,g). In agreement with the rheology data, statistical analysis showed a significant effect of polymer concentration on the compressive and tensile moduli (Figure 6f,h). The compressive modulus of 15% bioadhesives (84 ± 4 kPa) was significantly lower than 20% (207 ± 9 kPa, *p* < 0.0001) and 20% UV (255 ± 25 kPa, *p* < 0.0001) samples, a difference of approximately 3-fold (Figure 6f). Despite this difference, all groups resisted 8 cycles of 60% strain without apparent damage, demonstrating the mechanical stability of GelMA bioadhesives. Additionally, our results showed that 15% samples (34 ± 9 kPa) exhibited lower tensile modulus than 20% (51 ± 6 kPa, *p* < 0.01) and 20% UV (60 ± 7 kPa, *p* < 0.001) adhesives (Figure 6h). Higher compressive and tensile moduli were observed for 20% UV samples when compared to 20%; however, only the difference in the compressive moduli was significant (*p* < 0.01). Finally, the ultimate tensile strength of 15, 20 and 20% UV bioadhesives was 36 ± 5, 50 ± 10 and 47 ± 10 kPa, respectively (*p* > 0.05, Figure 6i), and the maximum elongation was between 130–150% (*p* > 0.05, Supplementary Figure S1).

### 3.4. Ex Vivo Retention of GelMA Bioadhesives

**Figure 7:**
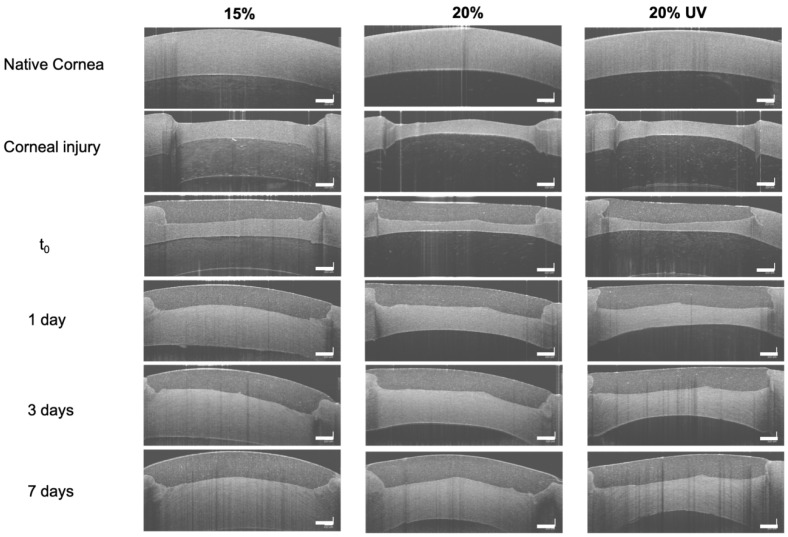
Ex vivo optical coherence tomography images of the native porcine cornea, partial-thickness corneal injury (⋍ 50% deep) and GelMA bioadhesives immediately after photocrosslinking (t_0_) and after 1, 3 and 7 days. Scale bar = 500 µm.

Next, ex vivo analysis was carried out to assess if the GelMA bioadhesive could fill a partial-thickness corneal injury and restore the corneal curvature. We observed that GelMA could be administered to the wound bed as a flowable liquid, fill the injury and adapt to the wound shape prior to physical crosslinking. As shown in Figure 7, GelMA bioadhesives formed a smooth contour on the corneal surface. The hydrogel remained optically clear after placement and exhibited excellent adhesion to the corneal tissue. After 24 h, stromal swelling could be observed due to absorption of water by the ex vivo cornea. Despite the increasing stromal pressure, all studied groups were retained in situ, without any signs of detachment, degradation, or shape loss at day 7.

### 3.5. In Vitro Assessment of Cytocompatibility

**Figure 8:**
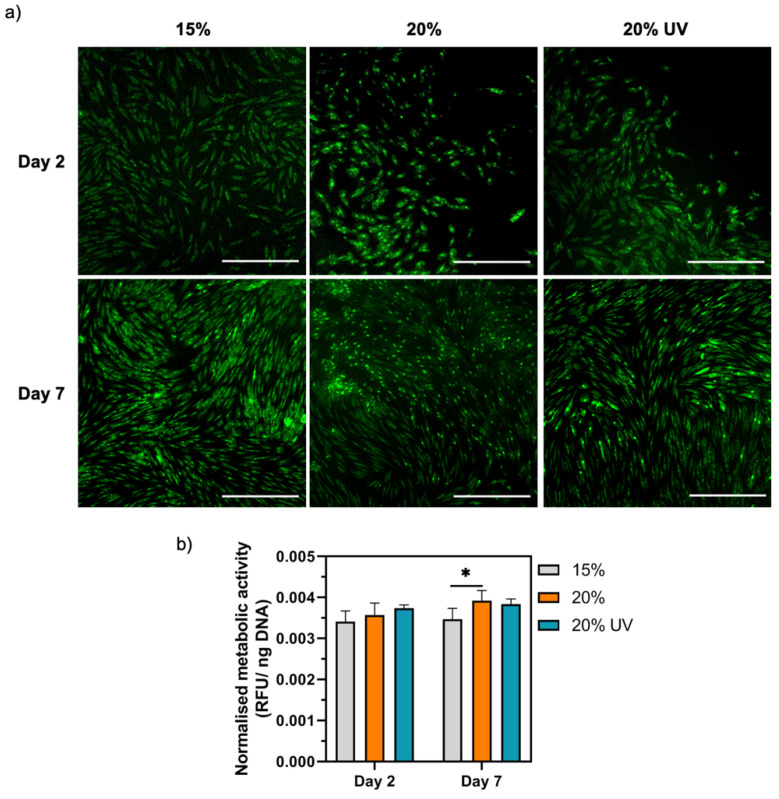
Cytotoxicity of GelMA hydrogels. (**a**) Live/Dead images of HDFs seeded on GelMA adhesives. Scale bar = 45 µm. (**b**) Normalized metabolic activity 2 and 7 days after cell seeding. Data are presented as mean value ± SD (* *p* < 0.05).

The viability, metabolic activity and proliferation of HDFs seeded on GelMA bioadhesives were evaluated. As shown in Figure 8a, live/dead images indicate high-cell viability for all tested conditions, with cells exhibiting a fibroblast-like morphology suggesting that GelMA bioadhesives did not induce toxicity. All groups were capable of supporting cell attachment and proliferation, with cell confluency being reached within 7 days. Since no dead cells were observed in the live/dead images, the proliferation of cells on the hydrogels was subsequently quantified. The DNA-normalised metabolic activity of HDFs cells seeded on 20% and 20% UV GelMA bioadhesives was slightly higher than 15% samples throughout the experiment (Figure 8b). However, the difference was only significant between 15 and 20% GelMA bioadhesives at day 7 (*p* < 0.05).

## 4. Discussion

Currently, there is a clinical need to develop strong and leak-free tissue adhesives that preserve corneal tissue function, decreasing postoperative morbidity and improving patient comfort [2]. While CA glues are toxic and associated with a range of complications, biocompatible fibrin and PEG-based adhesives are weak and degrade quickly making them ineffective to be applied as stromal fillers and for long-term treatments. This study aims to engineer a biocompatible and optically clear GelMA-based stromal filler with the adequate mechanical and adhesive properties to seal full-thickness corneal perforations. 

Ease of use and rapid gelation times are essential characteristics for the clinical suitability, acceptance and success of an adhesive. To achieve fast gelation (≤2 min), the photosensitive GelMA prepolymer solution was mixed with RF and SPS. As shown in Supplementary Figure S2, RF is a naturally occurring photosensitive molecule with a distinct absorption peak in the UV (λ=375 nm) and visible (λ=450 nm) range, which have high molar extinction coefficients (Supplementary Table S1). Photoinitiators with lower extinction coefficients would require longer irradiation times or higher photoinitiators/accelerators concentrations that are incompatible with the desired application given the increased photocrosslinking time and possible cytotoxic effects. In addition, the fact that both visible and UV can trigger the free-radical crosslinking reaction allows the photocrosslinking capability testing of both wavelengths. Since light is a form of electromagnetic energy, depending on its intensity, both UV, visible and infrared light can cause ocular damage [60]. Nevertheless, several ocular structures absorb or scatter some of the light entering the eye, protecting the retina from damage. In this study, the UV wavelength (365 nm) and riboflavin (2.66 mM) concentration were chosen in agreement with CXL therapy [54]. Li et al. used UV radiation (365 nm, 35 mW/cm^2^, 2 min) to crosslink a thiol-acrylate hydrogel on a rabbit’s cornea. Post-implantation histological examination did not find any signs of inflammation or tissue damage on the corneal epithelium, stroma or endothelium [13]. Importantly, the photocrosslinking parameters used by Li et al. were very similar to the conditions used in this study to cure 20% UV GelMA (365 nm, 30 mW/cm^2^, 2 min). In this study, the photocrosslinking capability of visible light (lower energy) was also assessed through the preparation of 15 and 20% GelMA bioadhesives. Lastly, RF’s slow gelation kinetics would be incompatible with the desired application [61]. To overcome this issue, we utilised SPS, an electron acceptor that is reported to enable the fast and safe photocrosslinking of polymers [61,62]. The 15, 20% and 20% UV GelMA bioadhesives were crosslinked under physiological conditions since all components are water soluble and the system RF/SPS is not susceptible to oxygen inhibition. Taken together, we showed the importance of employing the appropriate photoinitiator/curing system on the utility of the corneal adhesive in the clinical setting. 

The burst pressure is the most relevant parameter to assess the efficacy of GelMA bioadhesives in sealing full-thickness corneal perforations under liquid pressure. Thus, the mechanical adhesion of GelMA formulations was assessed in porcine eyes and compared with Histoacryl (CA). CA adhesives are proinflammatory, induce corneal vascularization and scarring, and inhibit corneal tissue regeneration [23]. Still, given its strong adhesion strength, fast polymerisation and ease of use, these adhesives are currently used ‘off-label’ by ophthalmologists to seal ocular wounds [18]. In this work, no significant differences in the burst pressure were observed between 20 and 20% UV GelMA samples and the control, even though 15% bioadhesives showed a significantly lower burst pressure than Histoacryl. GelMA bioadhesives could withstand on average an IOP between 33 and 49 kPa without leakages, values that are at least 17-fold higher than the human physiological IOP range (2 ± 0.4 kPa, Figure 2f) [63]. Recently, several different photoinitiator systems have been combined with GelMA, drastically affecting the final properties of the hydrogels [11,13,34,35,36,37,43,45,46,47,48]. As shown in Supplementary Figure S3, the ocular burst pressure obtained using LAP (~11 kPa) was around 4-times lower than RF/SPS (~48 kPa, *p* < 0.0001), highlighting the advantages of using this system when crosslinking GelMA hydrogels. 

After surgery and during the healing process the adhesives will be subjected to shear forces due to eyelid and eyeball movements. This will likely cause patient discomfort and potentially result in implant failure. Therefore, we evaluated the physical robustness of our GelMA bioadhesive in satisfying these critical parameters. We employed the in vitro lap shear tests to investigate the adhesive’s performance on wet and dry substrates. The average shear strength of GelMA bioadhesives was higher than Histoacryl, which was only not significantly lower than 20% GelMA (Figure 3b). Moreover, GelMA achieved strong adhesion in dry (gelatine-coated glass slides), semi-wet (porcine cornea mounted on ACC) and wet (full-thickness injury in eye) substrates, while Histoacryl was only effective to glue wet substrates. This highlights the versatility of GelMA bioadhesives and supports their suitability to be applied in dynamic in vivo conditions. 

CAs are reported to fail in adhering to wet surfaces due to their solidification immediately upon exposure to water [36]. The moisture on the ocular surface needs to be carefully controlled and the adhesive rapidly applied, as it might polymerize before entering in contact with the wound bed. Due to this fast (10–60 s) but uncontrolled polymerization, together with a very low viscosity, the successful application of CA glues remains a challenge and specialised training is required [18]. Nonetheless, fast sealing is of utmost importance as it avoids glue dilution due to continuous fluid egress from the wound. In this study, once the GelMA bioadhesive is heated to 37 °C, the flowable polymer can be added to the ocular injury. After filling the wound bed, the solution will physically crosslink upon cooling since the ocular temperature is around 32 °C, forming an adhesive plug within seconds. Then, this reversible seal can be chemically crosslinked with light. 

As shown in Supplementary Figure S4b, in addition to fail as a stromal filler, Histoacryl also creates a rough and opaque seal that precludes vision and makes the visualisation of infectious infiltrates and wound healing assessment challenging (Figure 2h) [4]. Usually, a bandage contact lens is often needed to improve patient comfort [18]. In contrast, GelMA adhesives formed a smooth contour and restored the ocular curvature (Figure 7). The 15 and 20% GelMA adhesives were macroscopically clear with a light-yellow colour after curing and exhibited a transmittance of ~63% (Figure 4a,b). This lower transmittance value was due to the RF being trapped within the gel absorbing light in the 400–500 nm range (Supplementary Figure S5). Since RF does not participate in the crosslinking reaction, the optical transmittance increases to ~85% after 2–3 h as RF diffuses out of the hydrogel. This value is higher than the reported human corneal transmittance (78–80%) [64,65]. Although the filler will suffer degradation and remodelling in vivo, the bioadhesive needs to have similar transmittance to the human cornea to replicate visual acuity. However, 20% UV bioadhesives were opaque with an average transmittance of ~64% (Figure 4a,b). We observed a 2-fold decrease in the porosity and a significant pore size decrease (~37%) when UV light was used to crosslink 20% GelMA, which are thought to cause light scattering, affecting transparency. Together, our findings indicate the 15 and 20% GelMA bioadhesives show suitable optical properties for corneal engineering applications. Further, as previously reported by Gorth et al., we observed a negative correlation between the porosity and pore size and the tensile and compressive moduli of the adhesive (Figure 6f,h) [66]. However, the pore structure did not influence the adhesive’s burst pressure (Figure 2d,f). 

Lastly, CA adhesives form a water-tight, but inflexible, bond to tissue, which makes its application inadequate for tissues with physiological movement. In the management of ocular perforations, CA glue patching is reported to be successful only when the perforation is smaller than 1 mm and, in 30 to 50% of the cases, the glue needs to be reapplied due to leaks [18,23]. Consequently, an ideal adhesive for corneal perforations needs to be tough, but pliable. Hatami-Marbini studied the dynamic shear properties of corneal stroma using oscillatory experiments, showing that corneal G’ and G’’ varied from 2 to 8 kPa and 0.3 to 1.2 kPa, respectively [67]. In this work, all formulations exhibited a G’ within the mentioned range (3.4 to 5.5 kPa); however, the G’’ of our hydrogels was out of range (~0.1 kPa). Importantly, the hydrogels showed a G’’ that was an order of magnitude lower than G’, confirming the viscoelastic behaviour of GelMA bioadhesives (Figure 6b,c) [68].

Whilst commercially available fibrin and CA-based adhesives are not approved for ocular use, some PEG-based sealants were granted approval for corneal incision sealing [69,70]. However, it is important to note that these adhesives have not been designed for long-term integration in the cornea. Despite being useful in reinforcing suture sealing, these materials are inappropriate to function as stromal fillers due to poor adhesion, high swelling rates and short retention times (few hours to a couple days). ReSure is the only FDA-approved sealant for intraoperative management of corneal incisions, while OcuSeal is the only option approved in Europe [69,71,72]. Since these sealants are composed by 89% of water, they form a smooth and lubricious surface, avoiding the use of a bandage contact lens for improved comfort [73]. However, they are reported to be sloughed off in tears within 1–4 days due to eyelid movement and PEG hydrolysis/enzymatic degradation [18,69,71,74,75,76]. Additionally, despite exhibiting lower swelling than other PEG-adhesives, the weight increase in ReSure is approximately 35% [72]. Hydrophilic hydrogels are 3D water-swellable networks. High swelling ratios weaken the mechanical properties, potentiate degradation and impact the overall shape of the adhesives, which might influence sealing and induce pressure on the surrounding tissues. GelMA adhesives showed high dimensional stability with an expansion between 7 and 10%, thereby reducing the risk of implant mismatch (Figure 4e,f). Additionally, all studied formulations exhibited water content values similar to the human cornea (72–85%) [77,78,79].

Strong adhesion to the host tissue and in situ retention are paramount for the success of a bioadhesive [28]. The ex vivo retention of GelMA bioadhesives was studied by making a 50% deep corneal injury in porcine corneas. The average thickness the porcine cornea is approximately 1400 µm, whilst the human cornea is on average 500–700 µm thick (Supplementary Figure S4a) [80]. OCT images show negligible bioadhesive degradation in PBS over the course of the experiment (Figure 7). Corneal swelling and loss of corneal transparency was observed after 24 h due to ex vivo absorption of large amounts of water by the stromal proteoglycans [81,82]. Importantly, 15 and 20% GelMA bioadhesives remained optically clear throughout the experiment. Despite the increased stromal swelling pressure, GelMA bioadhesives remained attached to the cornea demonstrating a strong tissue bond. 

The GelMA adhesive strength depends on its cohesion (crosslinking density) and interfacial adhesiveness to the wound bed [28]. Such strong adhesion to the tissue is expected to arise from a synergistic effect between physical interaction (mechanical interlocking to the ECM), covalent bonding, hydrogen bonding and electrostatic and hydrophobic interactions [33,37,38]. As previously reported, the methacrylic/methacryloyl groups on GelMA can react with the thiol and amine groups on the tissue, imparting tissue adhesion due to the formation of chemical bonds between the two surfaces [46]. Furthermore, functional groups on GelMA backbone (e.g., –OH, –NH_2_) can also form hydrogen bonds with the corresponding component on the tissue interface [30]. Taken together, our results show that GelMA adhesives are dimensionally stable and restore the corneal curvature, while showing a strong adhesion and retained transparency in vitro. 

As with CAs, the rapid and uncontrollable polymerisation of ReSure and OcuSeal works both in favour and against them. Since these sealants polymerise within 10–20 s after mixing, a late application might result in polymer hardening within the applicator [33]. Conversely, if the adhesive is applied too early, the wound will not be properly sealed due to the low viscosity of the material [73,75]. Before exposure to light, GelMA forms a plug within the wound, but the adhesive is soft and can still be easily removed. The use of light to crosslink GelMA bioadhesives hardens the polymer and allows tuning over the polymerization rate depending on the desired mechanical properties (chemical crosslinking). 

The synergy between the mechanical and biological adhesive characteristics is of utmost importance in the adhesive’s success by preventing corneal scarring and vascularisation. Consequently, the absence of cell adhesion moieties in CAs and PEG-based adhesives halts its application as stromal fillers since cell adhesion is an essential step for successful tissue regeneration [37,72]. In this work, a protein-based polymer was chosen due to its biocompatibility, biodegradability and presence of cell adhesion sites (e.g., RGD sequences). Albeit having a negligible degradation in PBS, a time-dependent degradation in the presence of collagenase II was observed, demonstrating the biodegradability of the GelMA adhesives (Figure 4g). The fact that the bioadhesive can be enzymatically degraded, allowing cells to remodel their environment, is essential for wound repair. According to our results, the engineered hydrogels were not toxic and could support the adhesion and proliferation of HDFs over 7 days, confirming the cytocompatibility of the adhesive (Figure 8). These results also confirm that the reagents used in the photocrosslinking reaction (RF/SPS) were not cytotoxic at the concentrations used in this study. Previous studies using similar concentrations of SPS reported that this molecule is rapidly consumed during the photocrosslinking reaction to levels that are not cytotoxic to cells (<20 µm) [34,61]. 

Recently, some authors have used the Eosin Y/TEA/VC system to photocrosslink GelMA (GelCORE) [45,48] and glycidyl methacrylate-modified gelatine (GELGYM) bioadhesive hydrogels [37]. Both adhesives supported cell adhesion and proliferation. GelCORE effectively sealed corneal defects and induced stromal regeneration and re-epithelization in a rabbit stromal defect model [45]. Sani et al. compared the burst pressures of ReSure and GelCORE (20% GelMA, Eosin Y, TEA, VC, 100 mW/cm^2^, visible light, 4 min) on 2 mm full-thickness injuries on rabbit eyes. The authors obtained a burst pressure of 30 ± 4 and 15 ± 6 kPa for GelCORE and ReSure, respectively [45]. In this study, the average burst pressure GelMA (visible light, 2 min) on a 2 mm full-thickness injury on a porcine cornea was 49 ± 9 kPa (Figure 2d). Shorter gelling times improve the clinical usability of these adhesives given the importance of rapid polymerisation on the performance and overall acceptance of tissue adhesives [45,48].

To our knowledge, this is the first study to investigate GelMA hydrogel formation via RF/SPS mediated photocrosslinking. We demonstrated that GelMA bioadhesives are effective on both wet and dry substrates, highlighting its versatility and potential to be applied in different physiological conditions. Considering that our optimised formulation (20% GelMA) showed an averaged burst pressure of 49 ± 9 kPa and knowing that the normal physiological and hypertensive blood pressures are approximately 8–19 and 40 kPa, respectively, we hypothesise that GelMA bioadhesives could meet the criteria for vascular sealants [36]. Moreover, GelMA adhesives could also be assessed as corneal stromal substitutes replacing the need for donor corneas in keratoplasty. Finally, the main limitation of GelMA bioadhesives is related with the usability, as both the polymer and RF/SPS need to be reconstituted in PBS and heated to 37 °C and an external light source is required to crosslink the bioadhesive. Due to the difficulty in replicating in vitro the ocular complex physiological environment, long-term experiments in an ex vivo ocular model or in vivo will be required to further evaluate the intrinsically linked physical and biological attributes of these photocurable bioadhesives, such as light effect in retinal toxicity, cytotoxicity, host response and the adhesive performance and degradation in the eye (ocular movements, blinking and tear film). The delivery of antibiotics, growth factors or anti-inflammatory agents from GelMA bioadhesives should be considered in the future since the biopolymer hydrophilic nature provides a solubilizing environment for the incorporation of many bioactive molecules. 

## 5. Conclusions

This work describes the synthesis and characterisation of a novel protein-based bioadhesive for the treatment of corneal perforations. The injectable bioadhesive, composed by GelMA and a riboflavin/sodium persulfate photoinitiator system, can be photocrosslinked in situ within 2 min using either UV or visible light. Ex vivo experiments in porcine eyes demonstrated complete sealing of full-thickness corneal perforations in the absence of sutures. The engineered adhesive has high translational potential due to its superior adhesion to both wet and dry substrates, outstanding physical properties, viscoelasticity and cytocompatibility in comparison to the commercially available options (e.g., fibrin glue, ReSure and cyanoacrylates). 

## Data Availability

The data presented in this study are available on request from the corresponding author.

## Supplementary Materials

The following are available online at https://www.mdpi.com/article/10.3390/bioengineering9020053/s1. Table S1: Comparison between different biocompatible photoinitiators: toxicity, water solubility and molar extinction coefficient at different wavelengths (365, 405, 450, 500 and 500 nm); Figure S1: Tensile mechanical testing of GelMA bioadhesives: elongation at the point of failure; Figure S2: Normalised absorption spectrum of riboflavin aqueous solution; Figure S3: Mean burst pressure of GelMA adhesives photocrosslinked with LAP and RF-SPS photoinitiator system; Figure S4: Central porcine corneal thickness distribution and comparison between an intact cornea and an ocular injury filled with GelMA adhesive and Histoacryl; Figure S5: GelMA hydrogels transmittance in the visible range.
