# Supplementary material for: Photocurable GelMA Adhesives for Corneal Perforations
Source: Bioengineering (Basel). 2022 Jan 28;9(2):53. doi: 10.3390/bioengineering9020053 (PMC8868637; doi:10.3390/bioengineering9020053)
Supplement: Supplementary file 1 [file bioengineering-09-00053-s001.zip › bioengineering-1509323-supplementary.pdf]

Molar extinction coefficient determination

The molar extinction coefficient (ε) is a measure of how strongly a substance absorbs light at a particular wavelength. The ε of various type I photoinitiators (LAP and I2959) and type II photoinitiators (RF, eosin Y and ruthenium) was measured in the 365- 550 nm range, using the Beer-Lambert Law:

$$\varepsilon\,(M^{-1}cm^{-1}) = \frac{A}{lc} \quad (1)$$

Where A is the absorbance (AU) at a given wavelength, l is the path length (cm) and c is the concentration (M). Several concentrations of the same photoinitiator were prepared and the absorbance measured using UV fused quartz cuvettes (Thorlabs, USA) in a microplate reader (Spark Multimode, Tecan, Switzerland). The ε at a given wavelength was obtained through the division of the absorbance versus concentration curve slope by the cuvette path length (1 cm). Each concentration was measured in triplicate. The ε at 365, 405, 450, 500 and 550 nm was calculated.

Table S1. Comparison between different biocompatible photoinitiators: toxicity, water solutibility and molar extinction coefficient at different wavelenghts (365, 405, 450, 500 and 500 nm).

Photoinitiator		Toxicity LC_{50} (mg/kg)	Water solubility (mg/mL)	Molar extinction coefficient (ε, M^{-1} cm^{-1})				
				ε_{365}	ε_{405}	ε_{450}	ε_{500}	ε_{550}
**Type I**	LAP	N/A	29.4	216	19	-	-	-
	I2959	2000 (rat oral)	1	2.97	-	-	-	-
**Type II**	Riboflavin	10000 (rat oral)	100	1013 8	6799	11792	1793	-
	Eosin Y	2344 (rat oral)	100	1813	2211	6112	50216	3042
	Ruthenium	1999 (mouse intravenous)	soluble	6127	9326	18524	2771	-

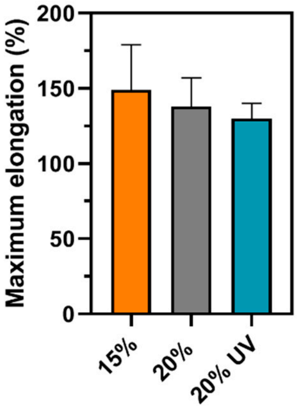

Figure S1. Tensile mechanical testing of GelMA hydrogels: elongation at the point of failure (%). Data presented as mean value ± SD.

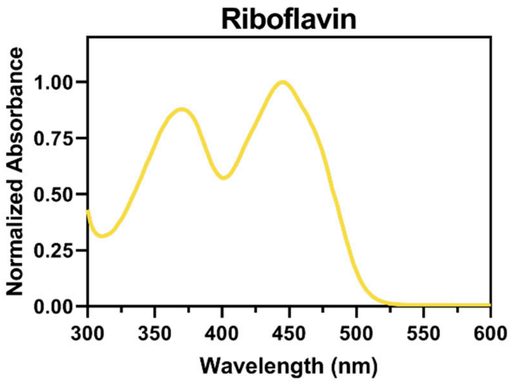

Figure S2. Normalized absorption spectrum of riboflavin aqueous solution.

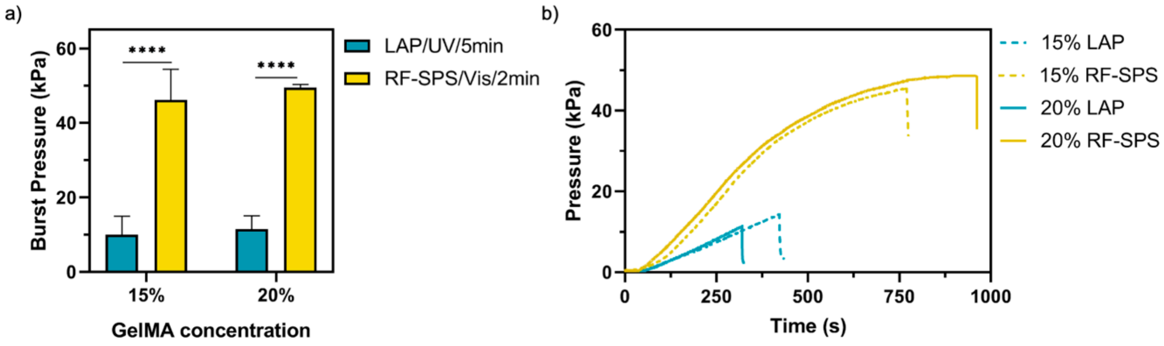

Figure S3. a) Mean burst pressure of GelMA adhesives photocrosslinked using two different photoinitiator systems in the artificial anterior chamber ($n = 8$): LAP (365 nm, 5 min) and RF-SPS (visible light, 2 min) ****$p<0.0001$. b) Representative pressure curves of the studied in the porcine eyeball.

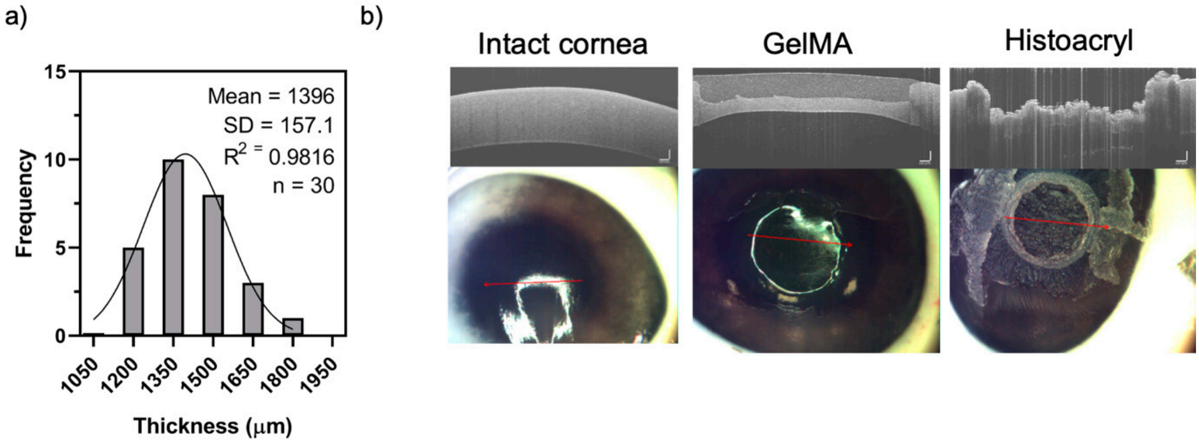

Figure S4. a) Central porcine corneal thickness distribution calculated from OCT images using ImageJ. b) Comparison between an intact cornea and an ocular injury filled with GelMA adhesive and Histoacryl. GelMA fills the injury by forming a smooth seal that restores the ocular curvature, while Histoacryl fails to fill the wound, creating an rough and opaque layer.

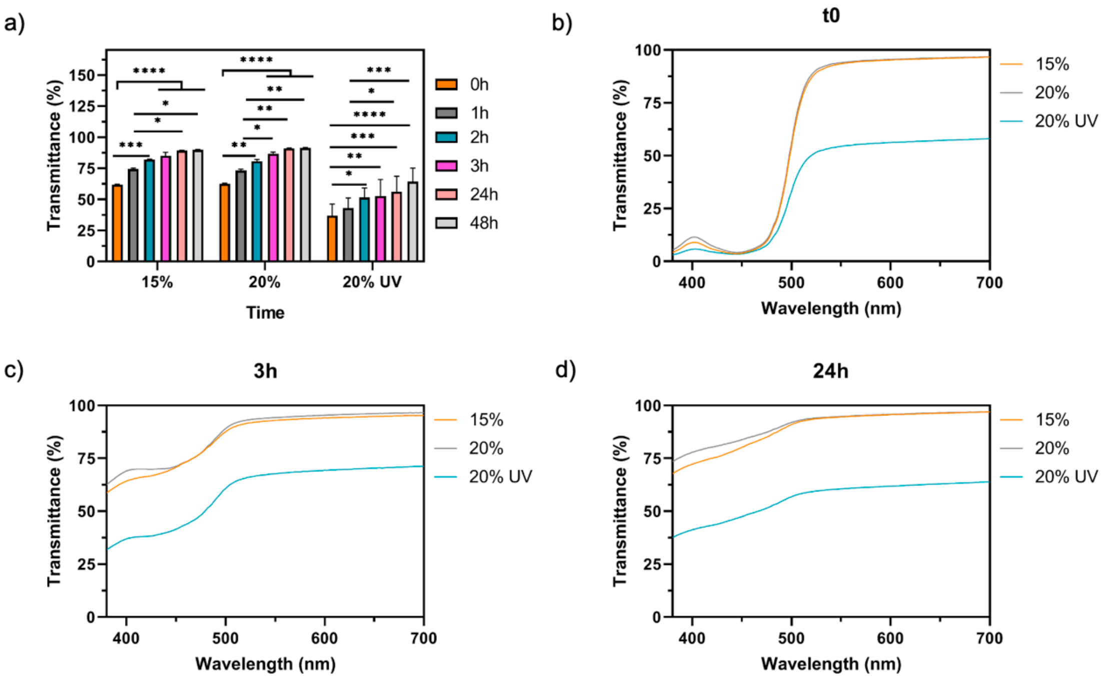

Figure S5. GelMA hydrogels transmittance in the visible range: a) average optical transmittance over time and optical transmittance profiles b) after curing (t_0) and c) after 3 h and d) 24 h incubation in PBS at 32°C. Data presented as mean value ± SD (*p<0.05, **p<0.01, ***p< 0.001, ****p<0.0001).
